# Study Clinical Characteristics and Outcomes of *Candida auris* Candidemia in Adult Intensive Care Unit Patients: A 30-Month Retrospective Study

**DOI:** 10.3390/jcm15166389

**Published:** 2026-08-18

**Authors:** Murat Unsel, Güldem Turan, Neval Elgormus, Hafize Uzun

**Affiliations:** 1 Department of Anaesthesiology and Intensive Care, Faculty of Medicine, Istanbul Atlas University, 34408 Istanbul, Turkey; 2 Department of Intensive Care Medicine, İzmir Tepecik Training and Research Hospital, University of Health Sciences, 35120 Izmir, Turkey; gturanmd@yahoo.com; 3 Department of Microbiology, Faculty of Medicine, Istanbul Atlas University, 34408 Istanbul, Turkey; neval.elgormus@atlas.edu.tr; 4 Department of Medical Biochemistry, Faculty of Medicine, Istanbul Atlas University, 34408 Istanbul, Turkey; hafize.uzun@atlas.edu.tr

**Keywords:** *Candida auris*, candidemia, intensive care unit, multidrug resistance, APACHE II, mortality, bloodstream infection, echinocandin

## Abstract

**Background**: *Candida auris* (*C. auris*) has emerged as a multidrug-resistant fungal pathogen causing healthcare-associated outbreaks worldwide, particularly in intensive care units (ICUs). Its environmental persistence, multidrug resistance, and high mortality make it a major challenge for infection prevention and clinical management. This study aimed to investigate the clinical characteristics, clinical exposures, treatment outcomes, and mortality of adult ICU patients with *C. auris* candidemia during the first 30 months after the opening of a tertiary care hospital. **Methods**: This retrospective observational study included all adult patients (≥18 years) admitted to five ICUs between 1 July 2020 and 31 December 2022 who developed culture-confirmed *C. auris* candidemia. Isolates were identified using matrix-assisted laser desorption ionization time-of-flight mass spectrometry (MALDI-TOF MS) with the Microflex LT/SH Smart MS system (Bruker Daltonics, Bremen, Germany) according to routine microbiological laboratory procedures. Demographic characteristics, underlying diseases, invasive procedures, clinical exposures, microbiological findings, treatment, APACHE II scores, and clinical outcomes were retrospectively analyzed. **Results**: During the 30-month study period, 3716 adult patients were admitted to the ICUs, and candidemia developed in 432 (11.6%). Of these episodes, 22 (5.1%) were caused by *C. auris*. The first *C. auris* candidemia case occurred 184 days after hospital opening. The mean age was 55 ± 17 years, and overall ICU mortality was 77.3%. Sepsis (36.4%), malignancy (22.7%), and cerebrovascular disease (18.2%) were the most common reasons for ICU admission. All patients had received broad-spectrum antibiotics, whereas central venous catheterization and invasive mechanical ventilation were present in 77.3% of cases. The mean interval from ICU admission to candidemia was 49 ± 15 days. Caspofungin was used as first-line antifungal therapy in all patients. Non-survivors had significantly higher APACHE II scores (32.8 ± 4.9 vs. 26.4 ± 6.6, *p* = 0.029) and predicted mortality rates (80.1% vs. 53.3%, *p* = 0.012). Antifungal treatment duration was significantly shorter among non-survivors (*p* < 0.001), whereas no significant differences were observed in age, ICU length of stay, or time to blood culture clearance. **Conclusions**: *C. auris* candidemia was observed in critically ill patients with prolonged ICU stays and frequent invasive interventions and was associated with high all-cause ICU mortality. Higher APACHE II scores were associated with mortality in this cohort, reflecting greater baseline severity of illness. Given the small sample size and descriptive, exploratory nature of the study, these findings should be interpreted with caution. The effectiveness of specific infection-control, diagnostic, or therapeutic interventions was not evaluated. Larger multicenter prospective studies are needed to better characterize the epidemiology, clinical outcomes, and factors associated with mortality in *C. auris* candidemia.

## 1. Introduction

*Candida auris* (*C. auris*), recently reclassified taxonomically as *Candidozyma auris*, is an emerging multidrug-resistant yeast that causes invasive infections and readily spreads among critically ill patients in healthcare settings. Although the updated taxonomic designation has been proposed based on phylogenomic analyses, the name *C. auris* remains the most widely used in the clinical and medical literature and is therefore used throughout this manuscript. *C. auris* has emerged as a multidrug-resistant fungal pathogen and is now recognized as a major cause of healthcare-associated outbreaks worldwide, particularly in intensive care units (ICUs) [1,2,3,4]. Since its first description in Japan in 2009, *C. auris* has spread rapidly across all inhabited continents owing to its remarkable environmental persistence, efficient person-to-person transmission, and ability to colonize both patients and healthcare environments [2,3,4,5].

Bloodstream infections caused by *C. auris* are associated with high mortality rates, ranging from 30% to 60%, particularly among critically ill patients with prolonged hospitalization, invasive medical devices, broad-spectrum antimicrobial exposure, and multiple underlying comorbidities [2,3,6]. Resistance to fluconazole is common, while reduced susceptibility to amphotericin B and echinocandins has also been increasingly reported, making treatment more challenging [2,4,6]. Consequently, the World Health Organization (WHO) has designated *C. auris* as a critical priority fungal pathogen because of its global public health importance [7,8].

The diagnosis of *C. auris* remains challenging because conventional biochemical identification systems may misidentify the organism, potentially delaying appropriate antifungal therapy and infection control measures. The implementation of matrix-assisted laser desorption ionization time-of-flight mass spectrometry (MALDI-TOF MS) has substantially improved the rapid and accurate identification of *C. auris* in clinical microbiology laboratories [2,3,6].

Several clinical factors have been associated with the development of *C. auris* candidemia, including prolonged ICU stay, invasive mechanical ventilation, central venous catheterization, renal replacement therapy, total parenteral nutrition, previous exposure to broad-spectrum antibiotics or antifungal agents, major surgery, diabetes mellitus, chronic kidney disease, malignancy, and immunosuppression [3,6,9]. Because these risk factors frequently coexist in critically ill patients, early recognition and prompt implementation of infection prevention strategies are essential.

Although the global incidence of *C. auris* continues to increase, data from Türkiye remain limited and are largely restricted to case reports and outbreak investigations [4,6]. Our tertiary-care hospital began admitting patients in July 2020, providing an opportunity to evaluate the early emergence of *C. auris* in a newly established healthcare facility.

Despite the increasing global spread of *C. auris*, published data from Türkiye remain limited, particularly regarding its early epidemiology and clinical characteristics in newly established healthcare institutions. To our knowledge, this is one of the first studies from Türkiye to comprehensively describe the epidemiology, clinical characteristics, clinical exposures, management, and outcomes of *C. auris* candidemia during the first 30 months after the opening of a newly established tertiary-care city hospital. By focusing on the early emergence of *C. auris* in a new healthcare setting, this study provides real-world epidemiological data that complement the existing literature and may contribute to future surveillance and infection-control strategies.

Because Candida auris has emerged as an important multidrug-resistant healthcare-associated pathogen characterized by rapid nosocomial transmission, environmental persistence, and high mortality, this study focused specifically on patients with culture-confirmed *C. auris* candidemia. Therefore, this study aimed to evaluate the clinical characteristics, treatment patterns, and outcomes of adult ICU patients with culture-confirmed *C. auris* candidemia diagnosed during the first 30 months after the opening of a newly established tertiary-care city hospital. We also sought to describe the clinical exposures observed in these patients and to provide real-world epidemiological and clinical data from one of the first comprehensive cohorts reported from Türkiye, thereby contributing to the limited national evidence on the early emergence of this multidrug-resistant pathogen.

## 2. Materials and Methods

### 2.1. Study Design and Setting

This retrospective observational study was conducted in the adult ICUs of a tertiary-care city hospital in Türkiye. The hospital started admitting patients on 1 July 2020. Adult patients hospitalized between 1 July 2020 and 31 December 2022, corresponding to the first 30 months after hospital opening, were screened for episodes of candidemia.

The study protocol was approved by the Institutional Ethics Committee (Approval No. 228; 7 June 2023) and was conducted in accordance with the ethical principles of the Declaration of Helsinki. Owing to the retrospective design and the use of anonymized clinical data, the requirement for informed consent was waived.

This study is reported in accordance with the Strengthening the Reporting of Observational Studies in Epidemiology (STROBE) Statement.

### 2.2. Study Population

Adult patients (≥18 years) admitted to one of the five ICUs during the study period who developed culture-confirmed *C. auris* candidemia were eligible for inclusion.

***Inclusion Criteria***

(i) Age ≥ 18 years; (ii) admission to an adult ICU; (iii) at least one peripheral blood culture positive for *C. auris*; (iv) availability of complete clinical and microbiological records.

***Exclusion Criteria***

(i) Patients younger than 18 years; (ii) duplicate bloodstream isolates obtained from the same infectious episode; (iii) incomplete clinical or microbiological records.

Candidemia was defined as the isolation of *Candida* species from at least one peripheral blood culture obtained from a patient with compatible clinical findings. For the epidemiological analysis, candidemia cases represented unique ICU patients. Duplicate positive blood cultures obtained during the same infectious episode were excluded, and only the first candidemia episode per patient was included in the analysis.

All episodes of *C. auris* candidemia occurred during ICU hospitalization and were considered healthcare-associated infections. No community-acquired cases were identified.

### 2.3. Microbiological Identification

Peripheral blood cultures were processed in the hospital microbiology laboratory according to routine laboratory procedures. Positive blood cultures yielding yeast isolates were subcultured on Sabouraud dextrose agar.

Yeast isolates were identified using matrix-assisted laser desorption ionization time-of-flight mass spectrometry (MALDI-TOF MS) with the Microflex LT/SH Smart MS system (Bruker Daltonics, Bremen, Germany) and the MALDI Biotyper Compass IVD version 4.2.90 database.

### 2.4. Treatment Protocol

Following microbiological confirmation of *C. auris* candidemia, all patients received intravenous caspofungin as first-line antifungal therapy, consisting of a 70 mg loading dose followed by 50 mg once daily, in accordance with current international treatment recommendations. According to the institutional treatment protocol, follow-up blood cultures were intended to be obtained every three days until microbiological clearance; however, because of the retrospective study design, follow-up blood culture results were not uniformly available for all patients. Antifungal therapy was generally continued for at least 14 days after the first documented negative blood culture, in accordance with current international recommendations and the treating physician’s clinical judgment.

### 2.5. Data Collection

Demographic, clinical, microbiological, and treatment-related data were retrospectively extracted from the electronic hospital information system and the microbiology laboratory database. The recorded variables included age, sex, primary indication for ICU admission, underlying comorbidities, APACHE II score and the corresponding predicted mortality rate, the interval between ICU admission and the development of candidemia, previous broad-spectrum antibiotic therapy, previous antifungal exposure, central venous catheterization, invasive mechanical ventilation, continuous renal replacement therapy, total parenteral nutrition, duration of antifungal therapy, time to documented blood culture clearance, and ICU outcome (survival or death). APACHE II scores were calculated during the first 24 h after ICU admission using routinely collected clinical and laboratory data, according to standard clinical practice.

### 2.6. Outcome Measures

The primary outcome was all-cause ICU mortality among patients with *C. auris* candidemia. Secondary outcomes included the interval between ICU admission and the development of candidemia, the duration of antifungal therapy, the time to documented blood culture clearance, and the distribution of underlying diseases and clinical exposures observed among patients with *C. auris* candidemia.

### 2.7. Statistical Analysis

Statistical analyses were performed using IBM SPSS Statistics for Windows, version 20.0 (IBM Corp., Armonk, NY, USA). The normality of continuous variables was assessed using the Shapiro–Wilk test separately within the survivor and non-survivor groups before between-group comparisons. Normally distributed continuous variables were presented as mean ± standard deviation (SD) and compared using the independent-samples *t*-test, whereas non-normally distributed variables were presented as median (interquartile range [IQR]) and compared using the Mann–Whitney *U* test. Categorical variables were expressed as frequencies and percentages and compared using the chi-square test or Fisher’s exact test, as appropriate. Time to documented blood culture clearance was summarized descriptively only among patients with documented clearance because follow-up culture data were not uniformly available. Given the limited sample size and the small number of survivors, all statistical analyses were considered exploratory and hypothesis-generating. Given the very small number of survivors (*n* = 5), the reliability of formal normality assessment was limited, and all between-group comparisons should therefore be interpreted cautiously. No adjustment for multiple comparisons was performed because of the exploratory nature of the analyses, and no multivariable prognostic model was developed. Accordingly, the results should be interpreted with caution. All statistical tests were two-sided, and a *p* value < 0.05 was considered statistically significant.

## 3. Results

### 3.1. Patient Characteristics

**Figure 1:**
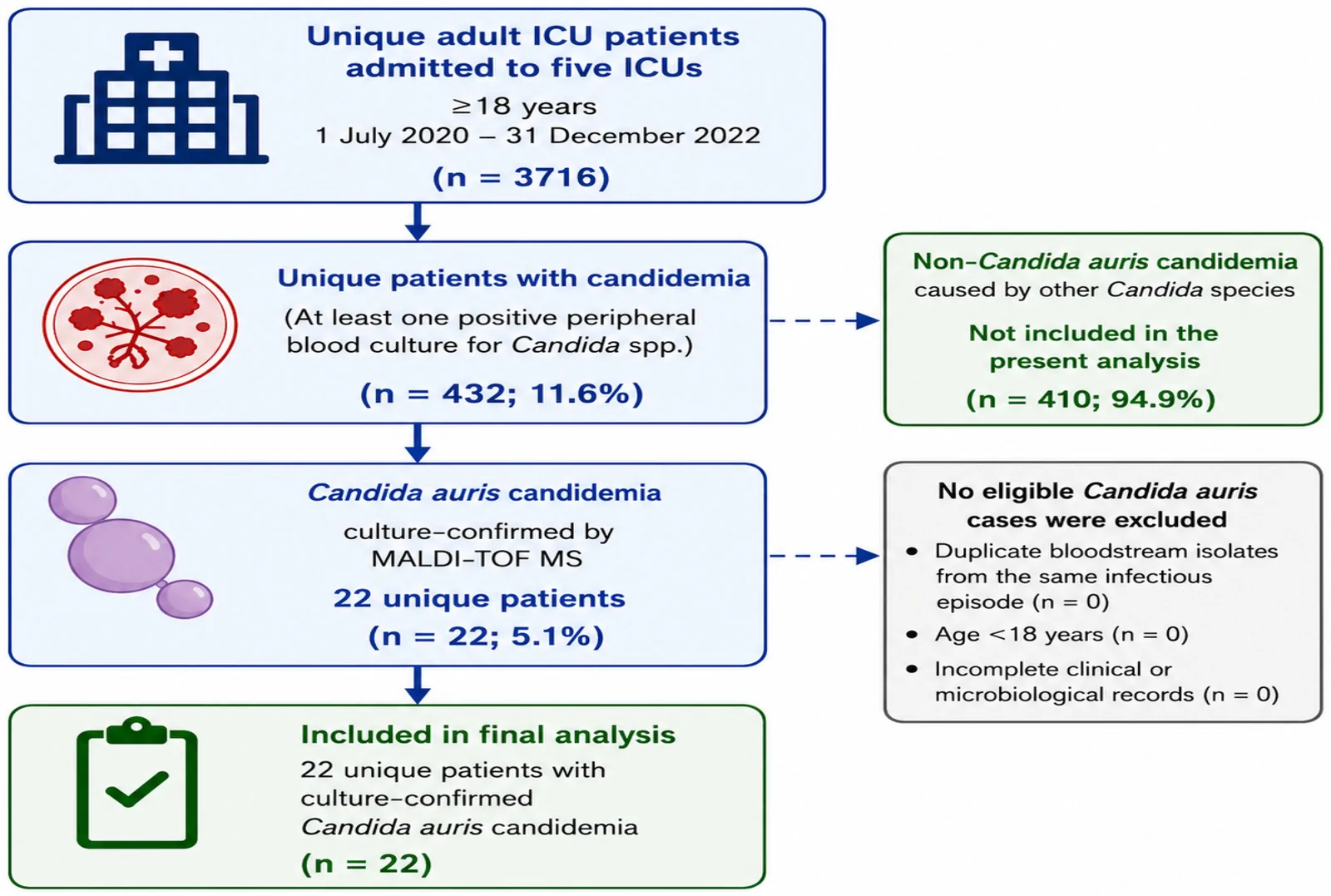
Flow diagram of patient selection. During the 30-month study period, 3716 unique adult patients were admitted to five intensive care units (ICUs). Candidemia, defined as the isolation of Candida spp. from at least one peripheral blood culture, was identified in 432 unique patients (11.6%). Among these, 22 unique patients (5.1%) had culture-confirmed *Candida auris* candidemia and were included in the final analysis. Only the first candidemia episode per patient was included in the final analysis, and duplicate bloodstream isolates obtained during the same infectious episode were excluded. ICU, intensive care unit; MALDI-TOF MS, matrix-assisted laser desorption ionization-time of flight mass spectrometry.

During the first 30 months following the opening of our tertiary-care hospital, 3716 adult patients were admitted to five ICUs (80 ICU beds). The patient selection process is summarized in Figure 1. Overall, 432 unique patients (11.6%) developed candidemia during ICU hospitalization, of whom 22 unique patients (5.1%) had culture-confirmed *C. auris* candidemia and were included in the study.

The first episode of *C. auris* candidemia was detected 184 days after hospital opening. The index case was a 47-year-old man admitted with an acute cerebrovascular accident and a medical history of diabetes mellitus and hypertension. He developed *C. auris* candidemia on the 68th day of ICU hospitalization.

The most common primary reasons for ICU admission were sepsis (8/22, 36.4%), malignancy (5/22, 22.7%), and cerebrovascular accident (4/22, 18.2%). Less frequent admission diagnoses included trauma (2/22, 9.1%), chronic obstructive pulmonary disease (1/22, 4.5%), liver cirrhosis (1/22, 4.5%), and cardiac arrest (1/22, 4.5%). All episodes of *C. auris* candidemia developed during ICU hospitalization and were therefore classified as healthcare-associated infections. No community-acquired cases were identified.

Accordingly, the clinical findings presented in this study reflect the characteristics of ICU-acquired *C. auris* candidemia rather than infection at the time of hospital admission.

### 3.2. Clinical Outcomes

The mean age of patients with *C. auris* candidemia was 55 ± 17 years. Survivors were younger than non-survivors (48 ± 19 vs. 57 ± 17 years), although the difference was not statistically significant (*p* = 0.305).

The mean interval between ICU admission and the development of *C. auris* candidemia was 49 ± 15 days, suggesting that most infections developed after prolonged ICU hospitalization. No significant difference was observed between survivors and non-survivors (*p* = 0.940).

The mean time to documented blood culture clearance among patients with available follow-up cultures was 11.1 ± 4.3 days (*n* = 14).

Among survivors, the mean duration of antifungal therapy was 21.8 ± 2.7 days. Treatment duration could not be reliably determined for non-survivors because complete treatment-duration data were not available; therefore, no between-group comparison was performed.

Mortality was evaluated as all-cause ICU mortality and was not attributed specifically to *C. auris* infection.

### 3.3. Disease Severity and Mortality

Disease severity was assessed using the APACHE II scoring system. Non-survivors had higher APACHE II scores than survivors (29.8 ± 4.1 vs. 23.2 ± 4.6, *p* = 0.006). Likewise, the predicted mortality rate was higher among non-survivors than among survivors (68.3 ± 12.2% vs. 46.6 ± 15.8%, *p* = 0.004). Given the small number of survivors, these between-group findings should be interpreted as exploratory.

Overall, 17 patients (77.3%) died during ICU hospitalization, whereas 5 patients (22.7%) survived to ICU discharge. The APACHE II score and corresponding predicted mortality rate were used as complementary measures of baseline disease severity and should not be interpreted as independent prognostic variables.

The reported mortality represents all-cause ICU mortality rather than mortality directly attributable to *C. auris* candidemia.

### 3.4. Primary Reasons for ICU Admission and Clinical Exposures

The primary reasons for ICU admission and the clinical exposures observed among patients with *C. auris* candidemia are summarized in Table 1. All patients (100%) had received broad-spectrum antibiotics before the onset of candidemia. Central venous catheterization and invasive mechanical ventilation were each present in 17 patients (77.3%), vasoactive agent use in 14 patients (63.6%), total parenteral nutrition in 10 patients (45.5%), previous systemic antifungal exposure in 10 patients (45.5%), corticosteroid therapy in 9 patients (40.9%), and continuous renal replacement therapy in 7 patients (31.8%). Because no comparison group was included, these findings describe the frequency of clinical exposures among patients with *C. auris* candidemia and should not be interpreted as independent risk factors for infection.

### 3.5. Comparison Between Survivors and Non-Survivors

The comparison between survivors and non-survivors is summarized in Table 2. Non-survivors had higher APACHE II scores and predicted mortality rates than survivors. The median duration of antifungal therapy was 20 (20–23) days among survivors and 10 (6–13) days among non-survivors (*p* < 0.001); however, this difference should be interpreted cautiously because treatment duration in non-survivors was truncated by death. Among patients with documented blood culture clearance, the median time to clearance was 6 (6–9) days in survivors and 12 (12–15) days in non-survivors. Given the very small number of survivors (*n* = 5), all between-group comparisons should be considered exploratory and interpreted cautiously.

## 4. Discussion

In the present retrospective study, we evaluated the clinical characteristics, clinical exposures, treatment outcomes, and mortality of patients with *C. auris* candidemia admitted to the ICU during the first 30 months after the opening of our hospital. The first case of *C. auris* candidemia was identified 184 days after the hospital became operational. This delayed occurrence may have been influenced by the infection prevention and control measures implemented from the beginning of hospital services. However, because no environmental surveillance, admission screening, or transmission analysis was performed, a causal relationship cannot be established.

Although *C. auris* infections have been reported across all age groups, they are more frequently observed in older adults, with the majority of cases occurring in individuals aged ≥60 years according to the Centers for Disease Control and Prevention (CDC) [10,11,12,13]. Cases have also been described in premature neonates [14]. Previous studies have not demonstrated a consistent sex predominance, although some reports have shown a slightly higher incidence among males [15]. In our study, patients who died were older than those who survived (57 ± 17 vs. 48 ± 19 years); however, this difference did not reach statistical significance (*p* = 0.305). Likewise, no significant difference in sex distribution was observed between the two groups, which is consistent with previous reports.

The proportion of candidemia observed in our ICU population was higher than that reported in many previous studies. This finding may reflect the characteristics of our tertiary-care referral center, which manages critically ill patients requiring prolonged ICU stays, invasive procedures, and extensive antimicrobial therapy. Nevertheless, comparisons across studies should be interpreted cautiously because candidemia frequency varies according to patient case-mix, surveillance methodology, and local epidemiology.

The mean time from ICU admission to the development of *C. auris* candidemia in our cohort was 49 ± 15 days, which is considerably longer than that reported in the literature. The CDC has reported that *C. auris* infections generally develop approximately 18–20 days after hospitalization, particularly among critically ill patients and those requiring invasive medical devices [13]. Similar findings have been reported from India, where candidemia developed after a mean of 19 days [16], and from New York, where symptom onset occurred around day 18 of hospitalization [17]. Conversely, outbreaks in Spain demonstrated that colonized or severely immunocompromised patients may develop infection within 7–10 days [18]. The prolonged interval observed in our study may be explained by the fact that all patients were managed in single isolation rooms with strict infection control precautions. Furthermore, our hospital was newly established, which likely reduced environmental contamination and cross-transmission. Recent studies have demonstrated that environmental contamination, patient colonization at admission, and healthcare-associated transmission play central roles in the emergence and persistence of *C. auris* outbreaks, highlighting the importance of active surveillance and environmental monitoring in healthcare settings [19]. Therefore, although the first *C. auris* case in our institution occurred 184 days after hospital opening, this finding should be interpreted cautiously because admission colonization screening, environmental sampling, and molecular transmission analyses were not performed. Consequently, the delayed occurrence cannot be directly attributed to the effectiveness of the implemented infection prevention and control measures.

All patients diagnosed with *C. auris* candidemia received echinocandin therapy according to institutional practice, consisting of a loading dose of caspofungin followed by maintenance treatment. Blood cultures were obtained every three days until microbiological clearance and treatment continued for at least 14 days after the first negative blood culture. According to CDC recommendations, blood cultures generally become negative within 4–7 days after initiation of appropriate antifungal therapy, although this period may extend to 10–14 days in resistant isolates [13]. In an Indian cohort, 65% of patients achieved blood culture clearance within five days, whereas resistant cases required 8–12 days [17]. In our study, the median time to documented blood culture clearance was 6 (6–9) days in survivors and 12 (12–15) days in non-survivors with documented clearance. The relatively prolonged time to documented blood culture clearance observed in this cohort may reflect the severity of underlying critical illness and prolonged ICU care; however, these findings should be interpreted cautiously because follow-up cultures were not uniformly available. The median duration of antifungal therapy was 20 (20–23) days among survivors and 10 (6–13) days among non-survivors (*p* < 0.001). The shorter duration of antifungal therapy observed among non-survivors most likely reflects treatment truncation due to early death rather than a therapeutic effect. The present study was not designed to evaluate the impact of antifungal therapy on the management of underlying diseases or treatment-related complications. Therefore, the shorter duration of antifungal therapy should not be interpreted as evidence that longer antifungal treatment improved survival.

Current international guidelines recommend echinocandins as the first-line therapy for *C. auris* candidemia, with treatment continued for at least 14 days after documented blood culture clearance [13]. Rudramurthy et al. [16] reported a mean treatment duration of 16–21 days among patients receiving echinocandin therapy. In our cohort, survivors received antifungal therapy for a mean duration of 22 ± 3 days, which was generally consistent with current treatment recommendations. The shorter duration of antifungal therapy observed among non-survivors should be interpreted cautiously, as treatment was inevitably truncated by death. Therefore, this finding most likely reflects reverse causation and survivor (immortal-time) bias rather than a therapeutic effect and should not be interpreted as evidence that longer treatment improved survival. Current international guidelines also emphasize prompt initiation of active antifungal therapy, appropriate source control, and evaluation for metastatic complications in patients with candidemia. However, adherence to these recommendations and their impact on clinical outcomes could not be evaluated in the present study [20,21].

Disease severity, as reflected by the APACHE II score, has been recognized as an important predictor of mortality in critically ill patients with *C. auris* infection. Previous studies have demonstrated significantly higher APACHE II scores among patients with *C. auris* candidemia than in general ICU populations, and scores greater than 20 have been associated with a three-fold increase in infection risk [16]. In a New York cohort, 72% of infected patients had APACHE II scores ≥ 25, and mortality reached approximately 60% among patients with higher scores [17]. A systematic review also concluded that elevated APACHE II scores increase the likelihood of developing *C. auris* infection by approximately 2.5-fold [14]. Consistent with these findings, non-survivors had higher APACHE II scores than survivors in our study (29.8 ± 4.1 vs. 23.2 ± 4.6, *p* = 0.006). Similarly, the predicted mortality rate was higher among non-survivors (68.3 ± 12.2% vs. 46.6 ± 15.8%, *p* = 0.004). These findings suggest an association between greater baseline disease severity and all-cause ICU mortality; however, given the very small number of survivors, these comparisons should be considered exploratory and interpreted cautiously. However, the observed ICU mortality should be interpreted as all-cause mortality rather than mortality directly attributable to *C. auris* candidemia. The higher APACHE II scores and predicted mortality rates observed among non-survivors are consistent with greater underlying critical illness severity in this group. Therefore, multisystem sepsis, cardiovascular dysfunction, and other organ failures were likely important competing contributors to mortality in addition to invasive fungal infection. This interpretation is supported by recent evidence demonstrating that sepsis-induced cardiovascular dysfunction is a major determinant of adverse outcomes in critically ill patients [22]. In addition, APACHE II and the corresponding predicted mortality are mathematically related measures; therefore, these findings should be interpreted as complementary indicators of baseline disease severity rather than independent prognostic variables. Because the present study evaluated all-cause ICU mortality, the observed deaths cannot be attributed solely to *C. auris* infection and were likely influenced by the severity of the underlying critical illness and associated comorbidities. The potential contributions of delayed diagnosis, antifungal resistance, or treatment-related factors could not be evaluated because these data were not consistently available in the present study.

The clinical exposures observed in our cohort were consistent with those reported in previous studies of *C. auris* candidemia. Commonly reported exposures include prolonged ICU stay, previous broad-spectrum antimicrobial therapy, central venous catheterization, invasive mechanical ventilation, and total parenteral nutrition [23,24].

Our findings should be interpreted as descriptive rather than causal because this study did not include a comparison group. Therefore, the frequencies of broad-spectrum antibiotic exposure, central venous catheterization, invasive mechanical ventilation, total parenteral nutrition, and continuous renal replacement therapy should be regarded as common clinical exposures among patients with *C. auris* candidemia rather than independent risk factors. Similar exposures have been identified as independent risk factors in analytical studies, including the Turkish case–control study by Kaya et al. [25] and the prospective international multicenter study by Erdem et al. [21]. The clinical exposures observed in our cohort were broadly consistent with those reported in these analytical studies. However, unlike these investigations, our study was not designed to evaluate causal associations or identify independent risk factors because no comparison group was included.

Consistent with these reports, our cohort demonstrated a high prevalence of broad-spectrum antibiotic exposure, central venous catheterization, invasive mechanical ventilation, and prolonged ICU hospitalization, together with substantial mortality. In contrast to these analytical studies, our investigation provides a descriptive overview of the clinical characteristics, treatment patterns, and outcomes of consecutive ICU patients with *C. auris* candidemia during the first 30 months after the opening of a tertiary-care hospital. Additionally, higher APACHE II scores were significantly associated with mortality, underscoring disease severity as an important prognostic indicator in this patient population.

In our study, sepsis was the most common reason for ICU admission (36.4%), followed by malignancy (22.7%) and cerebrovascular accident (18.2%). All patients had received broad-spectrum antibiotics, while 77.3% underwent central venous catheterization and invasive mechanical ventilation. Vasoactive agent administration was required in 63.6% of patients, total parenteral nutrition in 45.5%, continuous renal replacement therapy in 31.8%, and prior antifungal therapy in 45.5%. These findings describe the clinical profile of patients with *C. auris* candidemia in our cohort but should not be interpreted as evidence of independent risk factors because of the absence of a control group.

The principal strengths and novelty of this study are the provision of real-world data on the early emergence of *C. auris* candidemia during the first 30 months after the opening of a newly established tertiary-care hospital in Türkiye. Given the limited published evidence from the country, these findings expand the current epidemiological knowledge and may inform future multicenter surveillance studies and infection-prevention strategies.

This study has several limitations. It was conducted at a single center with a relatively small sample size and a retrospective design. Because no comparison group was included, the study was not designed to identify independent risk factors for *C. auris* candidemia but rather to provide a descriptive characterization of the clinical features and exposures observed among affected patients. Consequently, the study is subject to potential selection bias and did not allow adjustment for potential confounding factors. Moreover, because detailed clinical and outcome data for patients with candidemia caused by other Candida species were not available, direct comparisons of mortality and clinical outcomes between *C. auris* and non-auris Candida candidemia could not be performed. Data on ICU patient-days, admission colonization screening, patient transfers, contact tracing, environmental surveillance, infection prevention and control practices, molecular epidemiology, and transmission dynamics were unavailable; therefore, the effectiveness of infection prevention measures and transmission routes could not be formally evaluated. The small number of survivors limited the statistical power of between-group comparisons, precluded reliable prognostic modeling, and all analyses should therefore be regarded as exploratory and descriptive. Consistent with current methodological recommendations, robust prognostic model development requires an adequate sample size, appropriate variable selection, assessment of collinearity, and external validation, which were not feasible in the present study [26]. In addition, no adjustment for multiple comparisons was performed because of the exploratory nature of the analyses and the limited sample size; therefore, the reported *p* values should be interpreted with caution. Follow-up blood cultures were not uniformly available, particularly among patients who died early during treatment, limiting the assessment of microbiological clearance. Likewise, comparisons of antifungal treatment duration should be interpreted cautiously because treatment was inherently truncated by death in non-survivors. Detailed data regarding the timing of antifungal therapy, treatment-related complications, source-control interventions (including central venous catheter removal), evaluation for metastatic candidiasis (e.g., endocarditis and endophthalmitis), and concomitant bacterial or fungal co-infections were not consistently available and therefore could not be analyzed. Furthermore, molecular typing, clade analysis, or whole-genome sequencing of *C. auris* isolates was not performed; therefore, the geographic lineage, clonal relatedness, and potential transmission patterns of the isolates could not be determined. Finally, because the primary outcome was all-cause ICU mortality, the individual contribution of *C. auris* infection, underlying comorbidities, and severity of critical illness to mortality could not be determined. Despite these limitations, this study provides valuable descriptive data on the epidemiology, clinical characteristics, clinical exposures, treatment, and outcomes of *C. auris* candidemia in critically ill patients in Türkiye.

This retrospective study evaluated the clinical characteristics, clinical exposures, treatment outcomes, and mortality of ICU patients with *C. auris* candidemia during the first 30 months after the opening of our hospital. Our findings demonstrated that *C. auris* candidemia developed after prolonged ICU hospitalization and was frequently observed among patients with broad-spectrum antibiotic exposure, invasive mechanical ventilation, central venous catheterization, vasoactive agent use, total parenteral nutrition, and continuous renal replacement therapy. In addition, higher APACHE II scores were significantly associated with all-cause ICU mortality. The delayed onset of candidemia observed in our cohort should be interpreted cautiously, as no admission colonization screening, environmental surveillance, or transmission analyses were performed; therefore, this finding cannot be directly attributed to infection prevention and control measures. Our findings should be interpreted as descriptive rather than causal and do not demonstrate that specific infection prevention strategies or antifungal therapy improved clinical outcomes. Although limited by its retrospective design, single-center setting, relatively small sample size, and the absence of a control group, this study provides valuable data on ICU-associated *C. auris* candidemia in Türkiye. Larger multicenter prospective studies incorporating appropriate control groups are warranted to better characterize the epidemiology, identify independent risk factors, and optimize the clinical management of this emerging multidrug-resistant pathogen.

## Figures and Tables

**Table 1:** Primary reasons for ICU admission and clinical exposures among patients with *C. auris* candidemia (*n* = 22). COPD, chronic obstructive pulmonary disease; ICU, intensive care unit.

Variable	*n* (%)
Clinical exposures	
Broad-spectrum antibiotic therapy	22 (100)
Central venous catheterization	17 (77.3)
Invasive mechanical ventilation	17 (77.3)
Vasoactive agent use	14 (63.6)
Total parenteral nutrition	10 (45.5)
Previous systemic antifungal therapy	10 (45.5)
Corticosteroid therapy	9 (40.9)
Continuous renal replacement therapy	7 (31.8)

Data are presented as *n* (%). ICU, intensive care unit; COPD, chronic obstructive pulmonary disease.

**Table 2:** Descriptive Comparison of Survivors and Non-survivors Among Patients with *C. auris* Candidemia.

Variable	Survivors (*n* = 5)	Non-Survivors (*n* = 17)	*p*
Age (years), mean ± SD	48.0 ± 18.9	57.2 ± 16.6	0.305
Male sex, *n* (%)	4 (80.0)	7 (41.2)	0.311
Time from ICU admission to candidemia (days), mean ± SD	49.2 ± 17.5	48.8 ± 14.2	0.940
APACHE II score, mean ± SD	23.2 ± 4.6	29.8 ± 4.1	0.006
Predicted mortality (%), mean ± SD	46.6 ± 15.8	68.3 ± 12.2	0.004
Time to documented blood culture clearance (days), median (IQR) *	6 (6–9)	12 (12–15)	0.322
Duration of antifungal therapy (days), median (IQR) †	20 (20–23)	10 (6–13)	<0.001

Data are presented as mean ± standard deviation (SD), median (interquartile range [IQR]), or *n* (%), as appropriate. Normality was assessed using the Shapiro–Wilk test separately within the survivor and non-survivor groups. Normally distributed continuous variables were compared using the independent-samples *t*-test, whereas non-normally distributed continuous variables were compared using the Mann–Whitney U test. Categorical variables were compared using Fisher’s exact test, as appropriate. Given the very small number of survivors (*n* = 5), all between-group comparisons should be considered exploratory and interpreted cautiously. * Time to documented blood culture clearance was calculated only among patients with available follow-up cultures and documented clearance. † Antifungal treatment duration among non-survivors was inherently truncated by death; therefore, this comparison should be interpreted cautiously because of potential survivor bias/reverse causation.

## Data Availability

The original contributions presented in this study are included in the article. Further inquiries can be directed to the corresponding author.
